# Application value of the FOCUS-PDCA cycle in nursing care for dysphagia in patients with cerebral infarction: a meta-analysis

**DOI:** 10.1055/s-0045-1809417

**Published:** 2025-06-17

**Authors:** Xiaojing Lu, Jiandong Zhang, Ranran Wang, Xiujuan Liu

**Affiliations:** 1 First Hospital of Qinhuangdao, Intensive Care Unit, Qinhuangdao Hebei, People's Republic of China.; 2 First Hospital of Qinhuangdao, Department of Cardiovascular Surgery, Qinhuangdao Hebei, People's Republic of China.; 3 First Hospital of Qinhuangdao, Emergency Intensive Care Unit Area 1, Qinhuangdao Hebei, People's Republic of China.

**Keywords:** Quality Improvement, Deglutition Disorders, Cerebral Infarction, Quality of Life

## Abstract

**Background:**

In recent years, there has been growing interest in applying quality improvement methodologies to healthcare processes. One such approach is the Find, Organize, Clarify, Understand, Select-Plan, Do, Check, and Act (FOCUS-PDCA) cycle.

**Objective:**

To evaluate the effectiveness of the FOCUS-PDCA cycle in the management of dysphagia in patients with cerebral infarction.

**Methods:**

We conducted a comprehensive literature search on the PubMed, Embase, Cochrane Library, Web of Science, China National Knowledge Infrastructure, and Wanfang databases for articles published up to October 31st, 2024, following the guidelines of the Preferred Reporting Items for Systematic Reviews and Meta-Analyses (PRISMA) statement. Randomized controlled trials and high-quality retrospective studies comparing the FOCUS-PDCA cycle with conventional care were included. The primary outcomes were swallowing function, quality of life, neurological and limb functions, and complication rates. Data were pooled using random-effects models, and heterogeneity was assessed using the *I ^2^* statistic.

**Results:**

We included 6 studies involving 638 patients. The FOCUS-PDCA group showed significant improvements in swallowing function (standardized mean difference [SMD] = 1.65; 95%CI: 0.08–3.21), quality of life (SMD = 2.16; 95%CI: 0.54–3.79), and neurological and limb functions (SMD = 1.03; 95%CI: 0.07–2.00) compared with the conventional care group. The FOCUS-PDCA approach significantly reduced complication rates (risk ratio = 0.48; 95%CI: 0.32–0.68).

**Conclusion:**

The FOCUS-PDCA cycle appears to be an effective strategy to improve outcomes in patients with cerebral infarction and dysphagia. However, more high-quality studies are needed to confirm these findings and guide clinical practice.

## INTRODUCTION

Cerebral infarction, also known as *ischemic stroke* , is a major cause of morbidity and mortality worldwide. 1 It occurs when blood supply to a part of the brain is interrupted, leading to tissue damage and neurological deficits. 2 One common and potentially life-threatening complication of cerebral infarction is dysphagia, or difficulty in swallowing, which affects up to 80% of the survivors of stroke. 3

Dysphagia impairs a patient's ability to eat and drink, and it increases the risk of aspiration pneumonia, malnutrition, and dehydration. 4 These complications can significantly impact patient outcomes, prolong hospital stays, and increase healthcare costs. 5 Therefore, effective management of dysphagia in patients with cerebral infarction is crucial to improve patient outcomes and quality of life.

In recent years, there has been growing interest in applying quality improvement methodologies to healthcare processes. One such approach is the Find, Organize, Clarify, Understand, Select-Plan, Do, Check, and Act (FOCUS-PDCA) cycle. 6 This systematic approach to continuous quality improvement has been widely used in various healthcare settings to enhance patient care and outcomes. 7

The FOCUS-PDCA cycle in dysphagia management for patients with cerebral infarction is a comprehensive, step-by-step approach designed to improve patient care. This methodical process begins with the identification of the problem of dysphagia in patients with cerebral infarction (find) and assembly of a team of healthcare professionals to address the issue (organize). The team then defines the current process of dysphagia management and its shortcomings (clarify) before analyzing the root causes of ineffective management (understand). Based on this analysis, appropriate interventions are selected to improve dysphagia care (select), and a detailed implementation plan is developed (plan). The team then implements the planned changes in dysphagia management (do) and evaluates the effectiveness of these changes (check). Finally, successful changes are standardized, and areas that need further improvement are identified (act). This cyclical approach ensures continuous quality improvement in dysphagia management for patients with cerebral infarction.

Although several studies have investigated the effectiveness of the FOCUS-PDCA cycle in the management of dysphagia in patients with cerebral infarction, the overall evidence remains fragmented. A comprehensive synthesis of available data is needed to guide clinical decision-making and healthcare policies.

The present meta-analysis aims to systematically evaluate the effectiveness of the FOCUS-PDCA cycle in improving the outcomes of patients with cerebral infarction and dysphagia. The study specifically seeks to assess the impact of FOCUS-PDCA on swallowing function, quality of life, neurological and limb functions, and complication rates. By synthesizing data from high-quality studies, the current meta-analysis aims to provide clinicians and healthcare administrators with evidence-based insights to inform the adoption of the FOCUS-PDCA cycle in dysphagia management for patients with cerebral infarction.

## METHODS

### Search strategy

A comprehensive literature search following the guidelines of the Preferred Reporting Items for Systematic Reviews and Meta-Analyses (PRISMA) statement was conducted across multiple databases to identify relevant studies on the effectiveness of the FOCUS-PDCA cycle in the management of dysphagia in patients with cerebral infarction published up to October 31st, 2024. The databases searched were PubMed, Embase, the Cochrane Library, Web of Science, China National Knowledge Infrastructure (CNKI), and Wanfang. The search terms used were: *FOCUS-PDCA* , *hospitalized patients with cerebral infarction* , *dysphagia* , and *cerebral infarction* . On PubMed, the following detailed search strategy was used: ( *Cerebral Infarctions* [Title/Abstract]) OR ( *Infarctions, Cerebral* [Title/Abstract]) OR ( *Cerebral Infarcts* [Title/Abstract]) OR ( *Anterior Choroidal Artery Infarction* [Title/Abstract]) OR ( *Subcortical Infarction* [Title/Abstract]) OR ( *Subcortical Infarctions* [Title/Abstract]) OR ( *Cerebral Infarction, Left Hemisphere* [Title/Abstract]) OR ( *Infarction, Cerebral, Left Hemisphere* [Title/Abstract]) OR ( *Left Hemisphere, Cerebral Infarction* [Title/Abstract]) OR ( *Infarction, Cerebral, Right Hemisphere* [Title/Abstract]) OR ( *Right Hemisphere, Infarction, Cerebral* [Title/Abstract]) OR ( *Right Hemisphere, Cerebral Infarction* [Title/Abstract]).

### Inclusion and exclusion criteria

Studies were included if they met the following criteria:

- Randomized controlled trials or high-quality retrospective analyses;
- Comparison of the FOCUS-PDCA intervention with conventional care for dysphagia in patients with cerebral infarction;
- Report of at least one of the following outcomes: swallowing function, quality of life, neurological and limb functions, and complication rates; and
- Full-text availability in English or Chinese.

Studies were excluded if they were non-comparative, did not focus on patients with cerebral infarction and dysphagia, or did not implement the FOCUS-PDCA cycle.

### Study selection and data extraction

Two independent reviewers screened the titles and abstracts of the identified studies. The full texts of potentially-eligible studies were then assessed for inclusion. Disagreements were resolved through discussion or consultation with a third reviewer. Data extraction was performed independently by two reviewers using a standardized form, with verification by a third reviewer to ensure accuracy and consistency. The extraction form included comprehensive information on the following:

- Study characteristics (author, year, country, study design, sample size, intervention duration, and follow-up period);
- Participant demographics (age, gender, stroke severity, and time since stroke onset);
- Intervention details (specific components of the implementation of the FOCUS-PDCA cycle and control group care); and
- Outcome measures (assessment tools, mean and standard deviation values, and event rates).

Discrepancies in the extracted data were resolved through consensus among the reviewers or adjudication by the third reviewer.

### Quality assessment

The quality of the studies included was assessed using the Newcastle-Ottawa Scale (NOS) for randomized controlled trials and retrospective analyses, which evaluates studies based on selection, comparability, and outcome assessment. Studies with scores ≥ 6 were considered high-quality.

### Statistical analysis

Meta-analyses were conducted using the RevMan 5.3 software (The Cochrane Collaboration). For continuous outcomes (swallowing function, quality of life, and neurological and limb functions), standardized mean differences (SMDs) with 95%CIs were calculated. For dichotomous outcomes (complication rates), risk ratios (RRs) with 95%CIs were computed. Heterogeneity among the included studies was first assessed using the *I ^2^* statistic, with values > 50% indicating substantial heterogeneity. Fixed-effect models were used when heterogeneity was low ( *I ^2^* ≤ 50%), whereas random-effects models were applied when substantial heterogeneity was detected ( *I ^2^* > 50%). Publication bias was evaluated using the Egger's test and funnel plots, when applicable.

## RESULTS

### Study selection and characteristics

**Figure 1:**
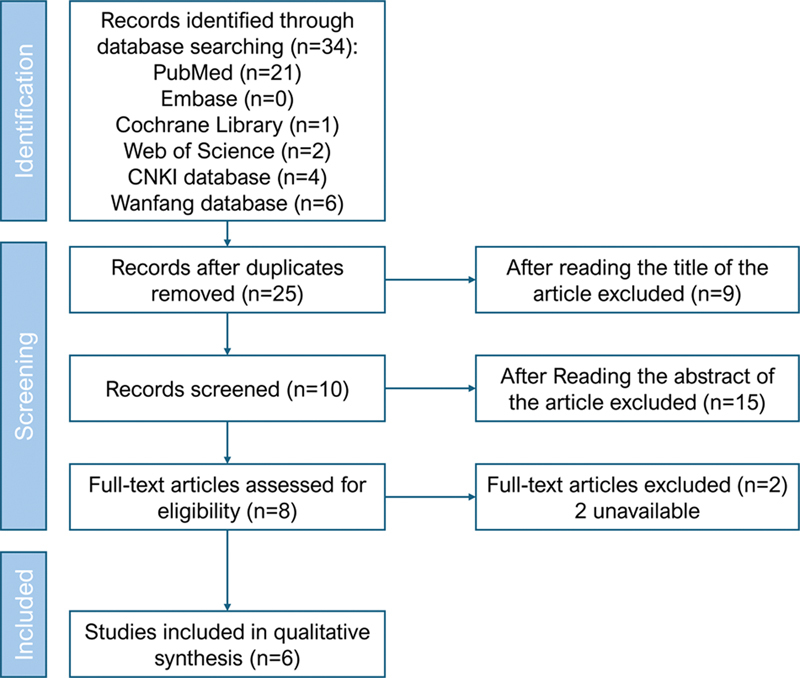
Flowchart of the study selection process.

In the initial database search, we identified 34 records (21 from PubMed, 4 from CNKI, 6 from Wanfang, 2 from Web of Science, and 1 from the Cochrane Library). After removing duplicates, 25 records remained for screening. Following the review of titles and abstracts, 10 articles were excluded, leaving 8 full-text articles for assessment. Ultimately, six studies 8-13 met the criteria and were included in the meta-analysis ( Figure 1 ).

The 6 studies included 8-13 involved a total of 638 patients, with sample sizes ranging from 42 to 234 participants. Five studies were randomized controlled trials, and one was a retrospective analysis. The intervention periods varied from 5 days to ∼ 16 days. The NOS scores of the studies included ranged from 6 to 7, indicating general high quality ( Table 1 ).

**Table 1:** Basic characteristics of included studies

Author (year)	Type of invention group	Type of study	Sample (trial group/control group)	Total sample	Intervention time	Exposure factors	NOs score
Wang and Zhou (2023) 8	FOCUS-PDCA	Randomized controlled trial	42/42	84	2 weeks	Swallowing function (VFSS), complication rate, quality of life (EDQOL)	7
Ji et al. (2016) 9	FOCUS-PDCA	Randomized controlled trial	47/47	94	2 weeks	Swallowing function (VFSS), efficacy of aspiration treatment, and incidence of stroke-associated pneumonia	6
Li (2018) 10	FOCUS-PDCA	Randomized controlled trial	43/43	86	5days/week, 1hour/day	LOTCA, BMI, andGQOLI-74 scores	7
Qu (2019) 11	FOCUS-PDCA	Randomized controlled trial	21/21	42	−	Efficacy of aspiration treatment and incidence of stroke-associated pneumonia	6
Zhang et al. (2009) 12	FOCUS-PDCA	Retrospective analysis	−	234	15.96 days	complication rate	6
Niu et al. (2024) 13	FOCUS-PDCA	Randomized controlled trial	49/49	98	−	Incidence rate of complications, swallowing function (VFSS), and neurological function and limb function	7

Abbreviations: EDQOL, Eating Disorders Quality of Life; FOCUS-PDCA, Find, Organize, Clarify, Understand, Select-Plan, Do, Check, and Act; GQOLI-74, Generic Quality of Life Inventory-74; LOTCA, Loewenstein Occupational Therapy Cognitive Assessment; MBI, Modified Barthel Index; NOS, Newcastle–Ottawa Scale; VFSS, videofluoroscopic swallowing study.

### Swallowing function

**Figure 2:**
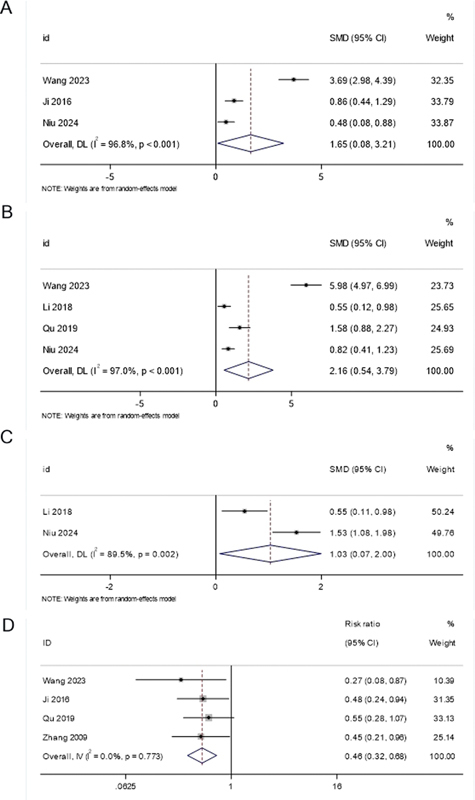
( **A** ) Forest plots for swallowing function (videofluoroscopic swallowing study, VFSS); ( **B** ) Forest plots for the quality of life; ( **C** ) Forest plots for neurological and limb functions; and ( **D** ) Forest plots for complication rate.

**Figure 3:**
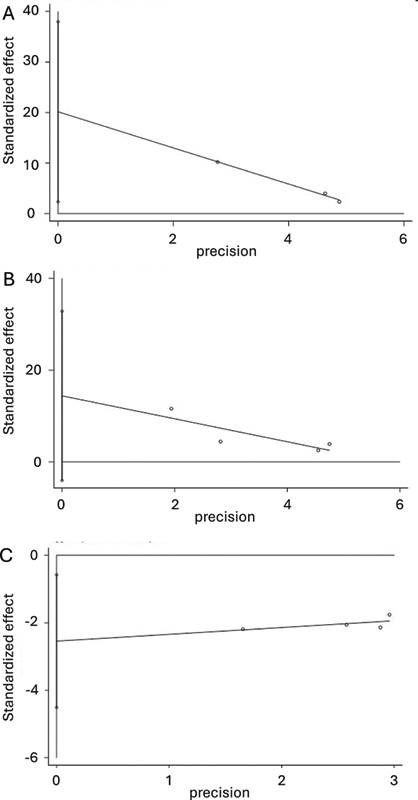
( **A** ): Egger's test plot for swallowing function (VFSS); ( **B** ) Egger's test plot for the quality of life; and ( **C** ) Egger's test plot for complication rate.

Three studies reported data on swallowing function using the videofluoroscopic swallowing study (VFSS). The meta-analysis revealed a significant improvement in swallowing function in the FOCUS-PDCA group compared with the conventional care group (SMD = 1.65; 95%CI: 0.08–3.21; *p* < 0.05). However, there was substantial heterogeneity among the studies ( *I ^2^* = 96.8%; *p* < 0.001). The forest plot for swallowing function is presented in Figure 2A . The Egger's test showed no significant publication bias ( *p* = 0.163; Figure 3A ). The funnel plot for this analysis appeared generally symmetrical.

### Quality of life

Four studies provided data on quality of life. The pooled analysis demonstrated a significant advantage for the FOCUS-PDCA group compared to the conventional care group in terms of quality of life (SMD = 2.16; 95%CI: 0.54–3.79; *p* < 0.05). Substantial heterogeneity was observed ( *I ^2^* = 97.0%, *p* < 0.001) among the studies. The forest plot for quality of life is shown in Figure 2B . The Egger's test indicated no significant publication bias ( *p* = 0.059.; Figure 3B ). The funnel plot for this analysis appeared generally symmetrical.

### Neurological and limb functions

Two studies reported on neurological and limb functions. The meta-analysis showed a significant improvement in neurological and limb functions in the FOCUS-PDCA group compared with the conventional care group (SMD =1.03; 95%CI: 0.07–2.00; *p* < 0.05). Substantial heterogeneity was observed between the two studies ( *I ^2^* = 89.5%; *p* = 0.002). The forest plot for neurological and limb functions is presented in Figure 2C . Due to the limited number of studies, the Egger's test for publication bias could not be performed for this outcome. The funnel plot for this analysis appeared generally symmetrical.

### Complication rates

Four studies reported on complication rates. The meta-analysis revealed a significantly lower complication rate in the FOCUS-PDCA group compared with the conventional care group (RR = 0.46; 95%CI: 0.32–0.68; *p* < 0.05). There was no significant heterogeneity among the studies ( *I ^2^* = 0.0%; *p* = 0.773). The forest plot for complication rates is shown in Figure 2D . The Egger's test indicated no significant publication bias ( *p* = 0.376; Figure 3C ). The funnel plot for this analysis appeared generally symmetrical.

## DISCUSSION

The current meta-analysis provides comprehensive evidence supporting the effectiveness of the FOCUS-PDCA cycle in the management of dysphagia in patients with cerebral infarction. The findings show significant improvements across multiple outcome measures, including swallowing function, quality of life, neurological and limb functions, and complication rates.

### Swallowing function

The substantial improvement in swallowing function (SMD = 1.65) observed with the FOCUS-PDCA approach is particularly noteworthy. This finding suggests that the systematic, iterative process of the FOCUS-PDCA cycle may be more effective than conventional care in addressing the complex nature of dysphagia in patients with cerebral infarction. The FOCUS-PDCA approach likely enables more targeted interventions, continuous monitoring, and timely adjustments to swallowing therapy protocols. 14

The significant improvement in swallowing function can be attributed to several factors inherent in the FOCUS-PDCA methodology. First, the *find* and *clarify* steps of the cycle encourage a thorough assessment of the current dysphagia management process, enabling healthcare providers to identify specific areas for improvement. This detailed analysis may lead to the discovery of previously-overlooked aspects of swallowing dysfunction or inefficiencies in the current treatment approach.

Second, the *understand* step promotes a deeper exploration of the root causes of dysphagia in patients with cerebral infarction. This comprehensive understanding can inform the development of more personalized and effective interventions. For example, healthcare providers may identify specific muscle weaknesses or coordination issues that contribute to dysphagia in individual patients, enabling targeted-therapy protocols.

The *select* and *plan* steps facilitate the choice and implementation of evidence-based interventions. This may include adopting novel swallowing techniques, using assistive devices or implementing specialized dietary modifications tailored to each patient's needs. The systematic planning process ensures that these interventions are implemented consistently and effectively across the healthcare team.

Furthermore, the *do* , *check* , and *act* steps promote continuous evaluation and refinement of the dysphagia management approach. This iterative process enables real-time adjustments based on patient responses and outcomes, potentially leading to more rapid improvements in swallowing function compared with static, conventional care approaches.

However, the high heterogeneity observed in this analysis ( *I ^2^* = 96.8%) warrants caution in interpreting these results. This heterogeneity may be due to variations in swallowing assessment methods, the specific components of the FOCUS-PDCA interventions or differences in patient populations across studies. Additionally, variations in how the FOCUS-PDCA cycle was implemented across different healthcare settings may have influenced the magnitude of the effect observed. Future research should aim to standardize swallowing function assessment and provide detailed descriptions of the implementation of the FOCUS-PDCA cycle to enhance comparability across studies.

### Quality of life

The significant improvement in quality of life (SMD = 2.16) associated with the FOCUS-PDCA approach is a crucial finding. Dysphagia can severely impact a patient's ability to eat, drink, and socialize, leading to reduced quality of life. 15 The emphasis of the FOCUS-PDCA cycle on continuous improvement and patient-centered care may contribute to addressing the multifaceted aspects of quality of life in patients with dysphagia. 16

The improvement in quality of life can be attributed to several aspects of the FOCUS-PDCA methodology. The patient-centered approach inherent in the FOCUS-PDCA cycle ensures that interventions are tailored to individual patient needs and preferences. This personalization may lead to greater patient satisfaction and engagement in the rehabilitation process, ultimately contributing to improved quality of life.

Moreover, the comprehensive nature of the FOCUS-PDCA approach may address not only the physical aspects of dysphagia but also its psychological and social implications. For example, the cycle may incorporate interventions to reduce anxiety related to eating in public or strategies to maintain social connections despite swallowing difficulties. By addressing these broader aspects of patient well-being, the FOCUS-PDCA approach may have a more profound impact on overall quality of life compared with conventional care focused primarily on physical symptoms.

The *check* and *act* steps also enable the regular assessment of patient-reported outcomes related to quality of life. This ongoing evaluation enables healthcare providers to promptly identify and address any emerging issues, potentially preventing long-term negative impacts on patient well-being.

Additionally, the team-based approach promoted by the FOCUS-PDCA cycle may lead to improved coordination of care across different healthcare disciplines. This integrated approach can ensure that all aspects of a patient's care, including psychological support and social services, are aligned to support an improved quality of life.

The substantial heterogeneity observed in this analysis ( *I ^2^* = 97.0%) may reflect the use of different quality of life assessment tools across studies or variations in the specific aspects of quality of life targeted by FOCUS-PDCA interventions. Additionally, patient characteristics such as age, comorbidities, and baseline swallowing function could significantly influence quality of life outcomes and contribute to heterogeneity. Future studies should consider using standardized, dysphagia-specific quality of life measures to enhance comparability and provide more nuanced insights into the impact of the FOCUS-PDCA cycle on patient wellbeing.

### Neurological and limb functions

The improvement in neurological and limb functions (SMD = 1.03) suggests that the benefits of the FOCUS-PDCA approach may extend beyond dysphagia management. This finding is particularly important given the interconnected nature of neurological recovery and swallowing function in patients with stroke. 17 The systematic approach to care of the FOCUS-PDCA cycle may facilitate better coordination between dysphagia management and overall stroke rehabilitation, leading to improved neurological outcomes. 18

The positive impact on neurological and limb functions can be attributed to several factors within the FOCUS-PDCA framework. First, the comprehensive assessment encouraged by the *find* and *clarify* steps may lead to a more holistic understanding of the patient's condition, including the interplay between swallowing function and overall neurological status. This broader perspective can inform the development of integrated rehabilitation strategies that address multiple aspects of recovery simultaneously.

Second, the *understand* step may promote a deeper exploration of the neurophysiological mechanisms underlying both dysphagia and broader neurological deficits in patients with cerebral infarction. This enhanced understanding can guide the selection of interventions that target shared neural pathways, potentially leading to synergistic improvements in swallowing and general neurological function.

The *select* and *plan* steps may facilitate the implementation of multidisciplinary interventions that address both dysphagia and broader neurological rehabilitation. For example, therapeutic exercises designed to improve oral motor control for swallowing may also have positive effects on speech production and facial muscle strength. Similarly, interventions aimed at improving overall trunk control and posture may enhance both swallowing safety and limb function.

Furthermore, the iterative nature of the FOCUS-PDCA cycle enables the continuous refinement of rehabilitation strategies based on observed outcomes. This adaptive approach may be particularly beneficial in addressing the complex and often non-linear recovery patterns observed in patients with stroke, leading to more effective neurological and functional rehabilitation.

The high heterogeneity observed between the two studies reporting this outcome ( *I ^2^* = 89.5%) highlights the need for further research in this field. Future studies should aim to elucidate the specific mechanisms through which FOCUS-PDCA interventions might impact neurological and limb functions in patients with cerebral infarction and dysphagia. Additionally, standardized assessment tools for neurological and limb functions should be employed to enhance comparability across studies.

### Complication rates

The significant reduction in complication rates (RR = 0.48) associated with the FOCUS-PDCA approach is a critical finding with direct clinical implications. Complications such as aspiration pneumonia are major concerns in dysphagia management and can considerably impact patient outcomes and healthcare costs. 19 The emphasis of the FOCUS-PDCA cycle on identifying and addressing potential sources of complications may contribute to this reduction in adverse events. 20

The lack of significant heterogeneity in this analysis ( *I ^2^* = 0.0%) suggests consistency in the effect of the FOCUS-PDCA cycle on complication rates across studies. This finding strengthens the reliability of the observed reduction in complications and supports the broader implementation of the FOCUS-PDCA cycle in the clinical practice for dysphagia management in patients with cerebral infarction.

### Limitations and future directions

Although the current meta-analysis provides valuable insights, several limitations should be acknowledged. First, the relatively small number of studies included ( *n* = 6) and the modest total sample size (638 patients) limit the generalizability of our findings. This limited sample size also prevented us from conducting meaningful subgroup analyses or meta-regression to explore the potential sources of the observed heterogeneity. Readers should interpret our results with caution, and future research should focus on conducting larger multi-center randomized controlled trials to provide more robust evidence.

Second, the studies included had relatively short follow-up periods, ranging from 5 days to ∼ 16 days. This variation in intervention duration may have influenced our findings, as longer interventions could yield different results compared with shorter ones. The inclusion of studies with such diverse intervention periods was necessary due to the limited available literature, but it introduced an additional source of variability to our analysis. Future research should investigate whether the duration of FOCUS-PDCA implementation affects patient outcomes and aim to establish optimal intervention periods for different patient populations.

Long-term studies are needed to assess the sustainability of the improvements observed with the FOCUS-PDCA approach and to evaluate its impact on long-term outcomes such as mortality and hospital readmission rates.

Third, the specific components and implementation methods of FOCUS-PDCA interventions varied across studies. Future research should aim to identify the most effective elements of the FOCUS-PDCA cycle in dysphagia management and develop standardized protocols to enhance reproducibility and comparability across studies.

Finally, although the present meta-analysis focused on clinical outcomes, future studies should consider cost-effectiveness analyses to provide a more comprehensive evaluation of the FOCUS-PDCA approach in dysphagia management for patients with cerebral infarction.

In conclusion, the current meta-analysis provides compelling evidence supporting the effectiveness of the FOCUS-PDCA cycle in the management of dysphagia in patients with cerebral infarction. Compared with conventional care, the FOCUS-PDCA approach demonstrated significant improvements in swallowing function, quality of life, and neurological and limb functions, while reducing complication rates. These findings suggest that the systematic, iterative process of the FOCUS-PDCA cycle may be particularly well-suited to address the complex and dynamic nature of dysphagia in patients with cerebral infarction.
